# Transglutaminase Crosslinked Milk Protein Concentrate and Micellar Casein Concentrate: Impact on the Functionality of Imitation Mozzarella Cheese Manufactured on a Small Scale Using a Rapid Visco Analyzer

**DOI:** 10.3390/foods13172720

**Published:** 2024-08-27

**Authors:** Prafulla Salunke, Lloyd E. Metzger

**Affiliations:** Department of Dairy and Food Science, Midwest Dairy Foods Research Center, South Dakota State University, Brookings, SD 57007, USA

**Keywords:** transglutaminase, milk protein concentrate, micellar casein concentrate, rennet casein, imitation mozzarella cheese functionality

## Abstract

In dairy-based imitation mozzarella cheese (IMC) formulations, intact casein is critical and imparts IMC with a firm and elastic, stringy, melted texture. Rennet casein (RCN) is the desired ingredient to provide intact casein in IMC and is preferred over milk protein concentrate (MPC) and micellar casein concentrate (MCC). Transglutaminase (TGase), a crosslinking enzyme, alters the physical properties of MPC or MCC and may change IMC functionality. The objective of this study was to determine the effect of TGase-crosslinked MPC and MCC powders on the functionality of IMCs. The TGase treatment included TGase at 0.3 (L) and 3.0 (H) units/g of protein and a control (C) with no TGase addition. Each IMC formulation was balanced for constituents and was produced in a Rapid Visco Analyzer (RVA). The MCC or MPC powder with high TGase enzyme in IMC formulation did not form an emulsion. The IMC containing TGase-treated powders had a significantly (*p* ≤ 0.05) higher RVA-viscosity during manufacture and transition temperature (TT), and a significantly (*p* ≤ 0.05) lower Schreiber melt test area. The IMC made from MPC (with or without TGase) had lower TT values and Schreiber melt test area as compared with that made from MCC. The TGase-treated MPC and MCC, when used for IMC manufacture, were comparable to IMC manufactured with RCN in texture and some measured melted characteristics. In conclusion, TGase treatment alters the melt characteristics of MCC and MPC in IMC applications.

## 1. Introduction

Imitation mozzarella cheese (IMC) is a misnomer for cheese analog, cheese substitute, or analog pizza cheese. They are categorized as either dairy-based or partially dairy-based [1,2,3]. There is another category called no dairy or non-dairy, wherein protein, fat, and other ingredients from non-dairy sources are used [1,2,3]. IMC is the primary application of analog cheese products in the United States (US), particularly on frozen pizza [3,4,5]. Some advantages of IMCs over natural cheeses are lower material and production costs, simple and uncomplicated manufacturing, and tailored functionality, including meltability, textural, nutritional, and compositional [1,2,3,4,5]. IMC is oil in water emulsion, where the protein and aqueous phase stabilize the oil phase. IMC manufacture requires blending and mixing various ingredients such as water, protein source, fat source, emulsifying salts, acidulant, flavorings, and preservatives, as well as heating and continuous mixing to produce an emulsion that sets once cooled [4]. The basic principles of IMC remain like those of processed cheese (PC) or processed cheese product (PCP) manufacture. Generally, PC equipment (a twin-screw augur cooker) is commonly used to make the IMC. This equipment mimics the mixing, blending, and stretching action of traditional mozzarella cheese manufacture [4,6,7,8,9,10]. Hence, the factors affecting PC functionality, such as the ingredients, processing, and equipment, are similar in IMC manufacture [11]. 

Intact casein, or unhydrolyzed casein, is the casein that has not undergone any breakdown or hydrolysis [2,8,9,11,12], except maybe rennet hydrolysis [12]. Typically, the protein ingredients used in IMC include rennet casein powder (RCN) or other high-protein ingredients such as acid casein [8,9,12,13,14,15], nonfat dry milk, skimmed milk powder, milk protein concentrates (MPC), and micellar casein concentrate (MCC) [8,9]. These ingredients not only provide total protein but also contribute to the significant amount of intact casein, forming a primary structural network of the IMC. The intact casein also plays a critical role in the unmelted textural [8] and melted [9] functional properties of IMC. 

Manufacturers of IMC prefer RCN to other protein sources because it provides desired unmelted and melted characteristics [8,9,11,13]. Casein in rennet casein is considered intact casein and provides higher viscosity on a protein basis when compared to other proteins [8,9,10,11]. The rennet casein is produced by coagulating skim milk with rennet and then isolating, washing, and drying the precipitated casein. The RCN is insoluble in water and has a high mineral content, particularly calcium and phosphorus. The high mineral content requires emulsifying salts to provide a firm texture and better viscoelastic properties in IMC or PCP. However, a wide variation in the quality and properties of RCN is available commercially, affecting the final quality of cheese analogs. Moreover, the US has limited production facilities for RCN manufacture. The imported RCN is utilized extensively in PC or IMC applications and has a severe economic impact on the cost of the finished product. There is a need to develop a product that is close to or superior to the available rennet casein because of quality (firm texture and good melt characteristics), availability, and economic considerations. Recently, acid casein with [14] and without [15] emulsifiers have been used to manufacture IMC. 

In the US, a substantial amount of MPC is currently manufactured [16], and its protein content ranges from 42% to 85%. Ultrafiltration (UF) is used to manufacture MPC (produce low lactose content) and high-protein ingredients with a concentration of casein and serum protein (SP) similar to the milk from which it was made [17]. However, in IMC formulation, the use of MPC is restricted due to serum protein. The serum or whey proteins denature at higher temperatures encountered during IMC manufacturing, causing product defects and a decrease in the functionality of IMC, as casein and serum (whey) proteins can interact at high temperatures. Hence, using MPC in formulation can result in undesirable characteristics such as a soft texture, reduced cooked viscosity, and limited melting characteristics [8,9,11,12,18,19]. In certain applications, micellar casein concentrates (MCC) are preferred over MPC in the formulation as MCC provides low SP. MCC is produced using a microfiltration (MF) process, which separates the SP from milk, and thus has higher intact casein compared to MPC. We found improved texture and viscosity of PCP produced with MCC compared with MPC [12]. This was a result of the decreased level of SP in MCC. Still, the cooked viscosity and the firmness of PCP produced using MCC were substantially lower than those manufactured using rennet casein. Unfortunately, MPC and MCC do not provide functionality similar to rennet casein in these applications [8,9]. 

In addition to excess whey protein, the texture and cooked viscosity issues of PC and IMC when elevated amounts of MPC or MCC are used in the formulation may be because of the κ-CN present on the surface of the casein micelle [8,9,12,18,19]. Since membrane separation techniques fractionate and concentrate the proteins in their native form, they are considered soluble and intact. The casein produced using membrane separation techniques is in micellar and native forms. The κ-CN and its GMP (glyco-macro peptide) fragment are present on the periphery of casein micelle, providing stability to the micelles via electrostatic repulsion of adjacent micelles. The GMP portion of κ-CN may interfere with the casein network formation in IMC because it is high in carbohydrates and has a strong surface negative charge on the casein micelle, causing defects such as reduced viscosity and soft texture. In contrast, the GMP portion of κ-CN is not present in rennet casein. The crosslinking enzyme can alter the physical and surface properties of casein, which may change the detrimental properties of κ-CN that are typically observed when MPC or MCC is used in IMC [8,9,12,18,19]. 

One of the methods available to alter the properties (structural and functional) of milk proteins is by crosslinking the proteins using crosslinking enzymes such as transglutaminase (TGase, EC 2.3.2.13). The TGase, after catalyzing the acyl transfer reaction between the glutaminyl residues (protein-bound) and the primary amines, gives rise to the formation of additional isopeptide bonds that impact the functional properties of dairy and food products. Hence, the use of the TGase enzyme has the potential to alter the physical properties of MPC or MCC and may change its functionality in IMCs [8,9]. Commercially, a twin-screw cooker is used to manufacture IMC, mozzarella cheese PC, PCP, or plant-based cheeses. Salunke et al. 2022a,b [8,9] evaluated the use of TGase-crosslinked MCC and MPC in IMC applications and compared it with the IMC made using RCN using a twin-screw cooker. They reported significant changes in unmelted and melted characteristics of IMC because of the TGase crosslinking of proteins. To evaluate and manufacture PC or PC-type products, a small-scale manufacturing protocol using a Rapid Visco Analyzer (RVA) has been developed [12,18,19,20,21]. RVA has been used to manufacture processed cheese [12,18,19,20,21,22,23], processed cheese food, and processed cheese products [12,18,19,24,25,26,27], processed cheese spread [28], model processed cheese [29,30,31] and rennet casein-based imitation cheeses [32], cheese spread [33] and model mozzarella cheese [34], or to analyze the melt characteristics [19,35]. Since the basic manufacturing protocols for PC, PCP, and IMC are similar, it was decided to use RVA to manufacture IMC. This study aimed to determine the effect of TGase-crosslinked MPC and MCC powders on the functionality of IMC manufactured in RVA and compared it with the IMC manufactured using rennet casein in RVA.

## 2. Materials and Methods

### 2.1. Experimental Design

**Figure 1:**
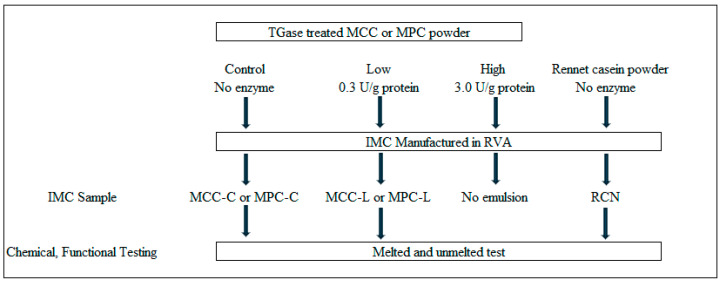
Experimental design.

The IMC in the RVA were manufactured using MCC and MPC and had three enzyme levels, namely control (no TGase, C), low (0.3 Units/g protein, L), and high (3.0 Units/g protein, H) in each (MPC or MCC). The experimental design is depicted in Figure 1. Initially, 2 × 3 factorial design experiments were set up with the type of product (MCC and MPC) and TGase enzyme amount (C, L, and H). However, in the preliminary trials, the MCC or MPC high TGase enzyme did not form IMC emulsion under any treatment and formulation combination; hence, it was dropped from this study. Finally, 2 × 2 factorial designs were set up for further studies with powders (MCC and MPC) and enzyme (C and L) amounts. The treatments were C-MCC (control—no enzyme MCC powder), L-MCC (Low enzyme MCC powder), C-MPC (control—no enzyme MPC powder), and L-MPC (Low enzyme MPC powder). Furthermore, commercially available rennet casein powder was used to manufacture IMC, serving as an additional standard control sample. The IMC manufactured using rennet casein was given code RCN. Each treatment was replicated thrice. 

### 2.2. Manufacture of Treatment Powders 

Each replicate of MCC using MF and MPC using UF was manufactured from pasteurized skim milk. The raw skim milk (South Dakota State University, Davis Dairy Plant, Brookings, SD, USA) was pasteurized (63 °C for 30 min) and immediately chilled to 4 °C. The skim milk (SM) was split into two equal batches. One batch was used for the manufacture of MCC using an MF unit, and another batch was used for MPC production using an UF unit. The process parameters of the experiment are described in our paper before [17]. The MCC and MPC retentate batches were again split into three equal lots and given TGase treatment. Two batches were treated with the required TGase (Activa TI, activity 100 units/g, Ajinomoto Food Ingredients LLC, Chicago, IL, USA), while the batch with no enzyme addition acted as control. Retentate after enzyme addition was incubated at 50 °C/25 min, then heated to 72 °C/10 min to denature the enzyme, and finally cooled to 4 °C. Control samples also followed similar steps. The blends were formulated for RCN (no enzyme) as reported in Table 1. The IMC sample manufactured using RCN acted as a second control sample. All the treatment retentates were dried in a spray dryer (Niro Inc., Columbia, MD, USA) to get treatment powders. Each treatment powder sample was stored separately in self-locking plastic bags at room temperature. The process details are given in detail in our paper published before [8]. The powders obtained were used as an IMC formulation ingredient.

### 2.3. Proximate Compositional Analysis

The proximate composition of IMC samples was determined using approved methods [36]. The IMC sample pH was measured using a spear-tip glass electrode (Accumet^®^ Fisher Scientific, Fair Lawn, NJ, USA) attached to a pH meter (340, Corning Life Science, NY, USA). 

### 2.4. Imitation Mozzarella Cheese Formulation and Manufacture

#### 2.4.1. IMC Formulation

The IMC was formulated and standardized using an Excel-based formulation software program (Techwizard, Owl Software, Columbia, MO, USA). The treatment powders (MCC and MPC) and commercial RCN were used as ingredients in respective IMC formulations and contributed to all the protein. The MCC powders had 3.1% moisture, 80.0% total protein (dry matter basis), and 4.5% lactose, whereas the MPC powders had similar moisture (3.1%), 76.1% total protein (dry matter basis), but higher lactose content (9.1%). These differences in lactose, protein, and even calcium content were due to differences in manufacturing processes [8]. The IMC was formulated to have moisture (48.0%), fat (21.0%), protein (20.0%), and salt (1.0%). The software balances various ingredients to give the required output. Hence, the RCN formulation required more lactose addition than treatment powders, as RCN had very low lactose. The formulation and the various ingredients used in the blend are given in Table 1. The fat source was butter (80.0% milk fat, unsalted, Great Value, Wal-Mart Stores, Inc., Bentonville, AR, USA). The emulsifying salt was sodium aluminum phosphate (Kasal, Innophos, Chicago Height, IL, USA). The formulation included other ingredients such as deproteinized whey powder (82.0% lactose, Davisco Foods International Inc., Le Surer, MN, USA), citric acid (KIC chemical Inc, New Peltz, NY, USA), lactic acid 85% *w*/*w* (Fisher Scientific, Fair Lawn, NJ, USA), and salt (iodized, Great Value, Wal-Mart Stores, Inc., Bentonville, AR, USA). For the rennet casein treatment, three different commercially available lots (Alaren^TM^ 771 edible RCN, New Zealand Dairy Board, New Zealand) were used. The RCN powder had an 80.3% total protein content, 10.2% moisture, and 0.1% lactose.

#### 2.4.2. IMC Manufacture

The MCC and MPC powders, each with two treatments and RCN as computed, were used in the preblend (200 g) of IMC formulation. The preblend of IMC was prepared by weighing all the ingredients and mixing (except lactic acid) in a blender (Kitchen Aid, St. Joseph, MI, USA) for 25 min before use. A Rapid Visco Analyzer (RVA-4, Newport Scientific, New South Wales, Australia) was used to manufacture IMC per the standard RVA protocol [19]. A 15 g portion of formulated preblend was weighed in the RVA canister along with water (0.5 g). The ingredients blend was mixed at 95 °C temperature using 1000 rpm for two minutes and additional mixing was done at 160 rpm/minute. However, since the enzyme treatment (L) in both products required additional heating, the heating temperature was increased to 3 min instead of 2 min. The lactic acid was added directly to the canister at the end stage of manufacture and mixed thoroughly before cooling. A total of 12 batches were produced from each preblend and used for various testing. The RVA-viscosity during manufacturing each replicate (12/treatment) of IMC was collected and then averaged. The IMC samples made in twelve batches were split into various subsamples for analysis. For texture profile analysis (TPA), five samples of IMC were transferred to copper molds (electroplated, 20 mm dia. × 30 mm height) immediately after manufacture in RVA. For the dynamic stress rheology (DSR) and modified Schreiber melt test, four IMC samples were transferred to plastic molds (30 mm dia.). All the samples were solidified at room temperature and transferred to the refrigerator to cool to 4 °C (overnight) before the analysis was completed. 

### 2.5. Functional Properties Analysis

#### 2.5.1. Textural Properties—Unmelted

The TPA was used for analyzing unmelted texture properties of IMC samples using TA.XT2 Texture Analyzer (Texture Technologies Corp., Scarsdale, NY, USA; Stable Microsystems, Godalming, UK). For TPA analysis, the copper molds (20 mm × 30 mm) filled during IMC manufacture were used, and samples were removed from the molds and cut to a height of 20 mm. The TPA was performed using uniaxial 2-bite compression standard protocol [8], and the TPA parameters were analyzed and calculated [8,37]. The tempering of samples was done at 20 °C temperature for 15 min. The test conditions used were a cylindrical flat probe (TA-25, 50 mm diameter) with a crosshead speed of 0.8 mm/s and sample compression of 80%. The test was replicated four times for each sample. 

#### 2.5.2. Textural Properties—Melted

##### Dynamic Stress Rheology (DSR)

For DSR analysis, the IMC samples were poured into a mold (30 mm diameter) soon after manufacture. IMC samples of 28.3 mm in diameter and 2 mm thickness were cut, put in the glass Petri plate, and covered (to avoid moisture loss). The samples were kept in the refrigerator at 4 °C until complete testing. A rheometer (Viscoanlyser, ATS Rheosystems, Rheologica Instruments Inc., NJ, USA) was used to measure DSR properties [9]. Temperature sweep was performed using parallel plate geometry (30 mm dia.) at a frequency of 1.5 Hz and the linear viscoelastic region constant stress of 400 Pa. To the rheometer’s upper plate, fine sandpaper (3M, 400 grits) was glued to avoid the sample sliding off. The vegetable oil (Crisco pure vegetable oil, The J M Smucker Co., Orrville, OH, USA) was applied on the exposed cheese surface to minimize drying of the sample while testing. The sweep was performed from 20 °C to 90 °C with temperature ramping at a rate of 1 °C/min. The elastic modulus (G′) and viscous modulus (G″) data were collected. The lowest temperature, where tan δ equaled 1 (G′ = G″), was designated as the transition temperature (TT) [9]. Each sample was tested in triplicate. 

##### Modified Schreiber Melt Test

IMC sample meltability was also measured using the modified Schreiber test [9]. Each IMC sample from the mold was cut into 28.5 mm (dia.) × 7 mm (height). The samples were tempered to 20 °C before the test. The four samples were placed on four different aluminum plates (0.95 mm thick; 100 mm × 100 mm), which were transferred immediately to an air convection oven (Fisher Scientific, NJ, USA) maintained at 90 °C and kept there for 7 min. The plates were removed from the oven and cooled to room temperature. Using a Vernier caliper, the diameter of the melted cheese was measured at 4 places for each sample, and the average was reported. The meltability of IMC was reported as the change in the area of the melted cheese in millimeters squared over the original area.

### 2.6. Statistical Analysis

The factorial design (2 × 2) with three replications was set up for statistical analysis with the product (MCC or MPC) and enzyme level (C and L) as main factors and the interaction term (product × enzyme level) using Proc GLM in SAS (Version 9.2, SAS Institute, Minato, Tokyo, 2009). ANOVA using main effects and interaction terms was prepared to obtain the *p*-values and mean squares. The comparisons of the mean value were made at a 0.05 level of significance using the least significance difference (LSD) and the results were considered significant at *p* < 0.05. Additionally, IMC made from RCN was also used as a control, and one-to-many comparisons were made using Dunnett’s test. Dunnett’s test was used for pairwise comparisons with a control.

## 3. Results and Discussion

### 3.1. Treatment Powder Composition and TGase Crosslinking

The proximate composition of treated MCC and MPC powders is described in our other published paper [8,9]. As per the authors, the MCC powders had higher proteins and calcium, whereas the lactose content was almost half, compared to MPC powders. These MCC and MPC powders’ moisture, fat, and ash content were comparable. The TGase treatment increased in the >50 kD protein fractions of the TGase-treated samples as measured by capillary gel electrophoresis [8] and particle size analysis [9]. As expected, the degree of crosslinking was greater in the treatment that utilized a higher TGase. The crosslinking effect was more pronounced in MPC than in MCC at higher TGase levels because the whey protein present in MPC caused inter-molecular crosslinking with caseins and increased molecular weight. However, the higher TGase level in MCC also significantly (*p* < 0.05) increased the small peptides (<10 kD) [8].

### 3.2. IMC Composition and Manufacture

#### 3.2.1. IMC Composition

The IMC formulation with a higher TGase amount did not form emulsion at any time-temperature combination, and hence was not used further. This may be because of the very high crosslinking and formation of the increased number of small peptides causing difficulty in emulsion formulation [8,9]. All the IMC formulations were balanced for proximate composition (Table 1). There were small differences in fat content of four treatment IMCs; however, no significant (*p* < 0.05) differences were observed in the pH, protein, and moisture content (Table 2). The differences in fat content among treatments were very small (range 0.05%) and did not affect overall results. The composition of IMC made using RCN and compared to treatment IMCs showed no significant (*p* < 0.05) differences. It should be noted that with the same TGase-treated powders using different compositions, ingredients, and emulsifiers, it was possible to manufacture PCP in RVA [18,19], indicating strong disruptive forces are needed for better emulsification. The high TGase powder did not form the emulsion even when a twin-screw cooker, which provides stronger shear and emulsification force, was used [8,9], indicating higher disruptive force is needed to break the strong covalent bond.

#### 3.2.2. IMC Manufacture

RVA collects the viscosity during the manufacture, and the RVA-viscosity is the viscosity at the end of the manufacture. During initial trials since the enzyme treatment (L) in both the products required additional heating, hence the heating temperature was increased to 3 min instead of 2 min to form a proper emulsion. The *p*-values and mean square values for the RVA-viscosity are depicted in Table 3. The enzyme level significantly (*p* < 0.05) affected the RVA-viscosity of IMC samples. The mean comparison showed that the IMC made using TGase treatment (MCC or MPC) had significantly higher RVA-viscosity compared to control samples without TGase (Table 4). The IMC sample made using C-MPC had significantly (*p* < 0.05) higher RVA-viscosity compared to C-MCC. This is because MPC has a high level of serum (whey) proteins, which denature at cooking temperature, causing extensive SP and CN interactions [8,9,11,12]. However, TGase addition increased RVA manufacturing viscosity significantly (*p* < 0.05) in both the products (L-MCC and L-MPC). There is a lack of literature available on the effect of TGase on RVA-viscosity during the manufacturing of IMC; however, in PCP formulation, the addition of TGase enzyme at a higher rate increased RVA-viscosity during manufacture [18,19,38].

### 3.3. IMC Functionality

#### 3.3.1. Unmelted Textural Characteristics

Unmelted textural parameters were measured in TPA including hardness (firmness), adhesiveness, cohesiveness, springiness, chewiness, gumminess, and resilience. Hardness is the most critical unmelted textural characteristic measured using TPA and describes the firmness of the IMC. The *p*-values and mean square values for the hardness are shown in Table 3. There was a significant (*p* < 0.05) effect of replication, enzyme level, and the interaction term on the hardness of the IMC samples. The significant (*p* < 0.05) interaction term suggests that changes were not linear. The enzyme level had a significant (*p* < 0.05) effect on all the TPA parameters except gumminess, whereas the product type significantly (*p* < 0.05) affected springiness, cohesiveness, chewiness, and resilience. Various TPA parameters average values are shown in Table 5. TGase treatment of MPC and MCC (L-MPC and L-MCC) significantly (*p* < 0.05) increased the springiness, gumminess, cohesiveness, resilience, and chewiness values compared to respective control samples (C-MPC and C-MCC), whereas it reduced adhesiveness values. This is because of the TGase crosslinking of protein. MPC had lower values of TPA parameters compared to MCC samples. A similar trend was reported when the IMC was made using a twin-screw cooker [8]. This suggests that RVA can be used to characterize ingredients before the plant runs quickly. 

The TPA-hardness (a measure of firmness) is the most important characteristic of IMC [8]. The firmness of IMC decides the machinability of cheese during the manufacturing, packing, shredding, sliceability, etc. The TPA-hardness of C-MPC was significantly low compared to the other three samples, indicating that the serum (whey) protein had a detrimental effect on hardness and produced IMC with a soft body. The TGase action in MPC improved the firmness of IMC samples, which was similar to MCC samples. IMC manufactured using a twin-screw cooker did show a similar trend in the hardness of TGase-treated IMC samples [8]. In the TGase-treated powder, the change in molecular weight of proteins indicating inter- and intra-molecular crosslinking by TGase enzyme has been reported to modify the surface properties of casein [8,38]. However, in PCP, the increase in firmness of TGase-treated samples has been reported [18,38].

#### 3.3.2. Melted Characteristics

**Figure 2:**
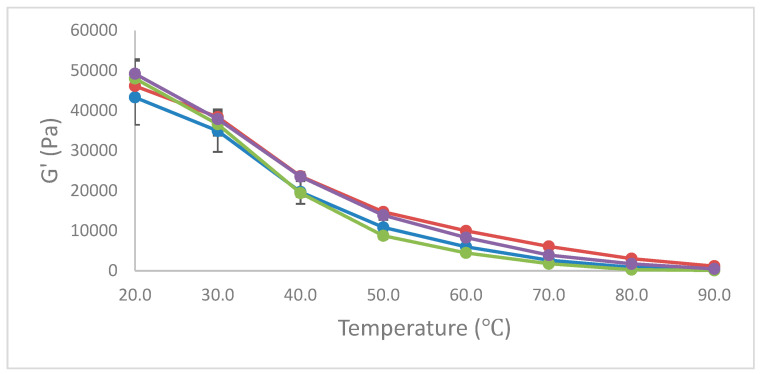

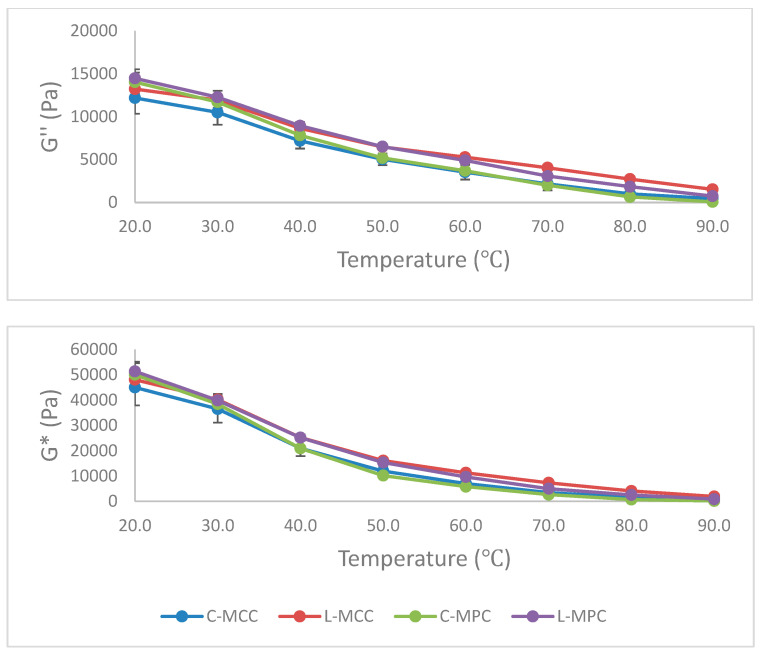
The melted functional properties (G′, G″, and G* values) of the 4 IMC ^1^ treatments manufactured in RVA ^2^. ^1^ IMC= imitation mozzarella cheese treatment; ^2^ RVA—Rapid Visco Analyzer. Imitation mozzarella cheese treatment: C-MCC = control—no enzyme, micellar casein concentrate powder; L-MCC = low enzyme, micellar casein concentrate powder; C-MPC = control—no enzyme, milk protein concentrate powder; L-MPC = low enzyme, milk protein concentrate powder; RCN = imitation mozzarella cheese treatment—rennet casein.

The melted functional properties in IMC are due to protein network and molecular interactions, and these post-manufactured melted characteristics of the IMC samples were measured using the Schreiber melt test and DSR (to obtain transition temperature). The *p*-values and the mean square values for the melted functional properties of IMC are given in Table 3. The type of product and level of enzyme had a significant (*p* < 0.05) effect on the Schreiber melt test and TT (Table 4). Figure 2 depicts the rheological properties G′ (elastic modulus), G″ (viscous modulus), and G* (complex modulus) of the four IMC treatments measured using DSR during temperature sweep (20–90 °C). The DSR properties (G′, G″ and G*) values of control IMC samples (C-MCC and C-MPC) were lower than that of samples treated with TGase (L-MCC and L-MPC). The C-MPC samples had lower G′, G″, and G* values compared to C-MCC samples above 50 °C. A similar tendency was observed for TGase-treated samples, where L-MPC had lower values of G′, G″, and G* compared to L-MCC samples. The DSR test gives an indirect interpretation of molecular-level interactions happening in the sample. The TGase treatment crosslinked proteins, modified protein structure, and changed the surface properties of casein micelles. The newly formed covalent bonds by TGase crosslinking are strong enough to maintain structure and resist the disintegration of structure [38].

The melt of the cheese is defined as the ability of cheese to flow upon heating and spread, along with the disappearance of the integrity of individual cheese shreds [39,40]. The PCP made from C-MCC had the highest change in melt area (modified Schreiber melt test). However, the addition of TGase caused a restriction in the flow and spread of cheese. Similarly, C-MPC had a higher melt area compared to L-MPC but lower than C-MCC, indicating that the serum (whey) proteins in MPC had a detrimental effect on melt characteristics. The addition of TGase caused the molecular level changes, causing restriction in the melt of the IMC samples. 

TT measures the initial melt characteristics of cheese to indicate molecular interactions and is used to evaluate the melt characteristics of IMC using DSR. The TT (tan *δ* (G″/G′) = 1), is a measure of the IMC melting point because this is the lowest temperature where a cheese changes from elastic to viscous [35,41]. This depends on protein structure and fat melting, and how it changes with the increase in temperature. The DSR-melt temperature of IMC made from C-MPC was the lowest while enzyme addition significantly (*p* < 0.05) increased the DSR-melt temperature in L-MPC. The PCP made from C-MCC had a similar DSR-melt temperature as of L-MPC, while TGase addition significantly (*p* < 0.05) increased the temperature to 82.1 °C. 

Overall, TGase enzyme addition significantly (*p* < 0.05) restricted melt as measured by melt area and TT. Melt characteristics depend on the casein interactions [40], and as expected, samples with high TT had low melt area and vice versa. Similar results were reported for IMC manufactured using twin screw cooker [9]. Crosslinking of proteins using TGase converts soluble milk proteins obtained through membrane separation techniques into insoluble proteins [8,9]. Other studies have also reported changes when TGase is used on changes in soluble to insoluble proteins [42,43], better emulsification [44], and the increase in stability against disruptive forces [44,45], which affect DSR values and TT. It has been reported that the enzyme concentration at higher levels makes caseinate gels more viscoelastic [46]. The crosslinking by TGase enzymes via covalent bond formation and modification of casein micelles surface caused restricted melt and higher TT. Similar results of restricted melt and increased TT in PCP manufactured using similar processes with TGase-treated powder have been reported [18,19,38]. 

### 3.4. Comparison with RCN Samples

A comparison of the RCN-manufactured IMC acting as a second control sample with the treatment sample IMC is given in Table 6. The RVA-viscosity of IMC manufactured using RCN was higher; however, the differences were non-significant when compared to TGase-treated IMC samples (L-MCC and L-MPC), whereas the differences were significant (*p* < 0.05) when compared to control samples (C-MCC and L-MPC). The main textural parameter hardness was slightly higher for RCN, but the differences were too small to be significant for all the treatment samples. The RCN and L-MCC were alike in almost all the TPA textural parameters, whereas minor but significant (*p* < 0.05) differences were seen when compared to L-MPC, C-MCC, and C-MPC samples. The IMC made using RCN had significantly (*p* < 0.05) lower TT (except C-MPC) and significantly (*p* < 0.05) higher change in the area compared to the treatment samples. This indicated that the TGase action and powder type (MPC and MCC) restricted melt in IMC compared to IMC manufactured using RCN.

The casein (intact) in the natural cheese or RCN powder is different from the native casein obtained during membrane separation or filtration (either UF or MF) [8,9,12,18,19,38]. Almost all casein micelle structure models have proposed that κ-CN covers the casein micelle surface and extrudes from the surface layer as a hair, possessing hydrophilic properties [47]. The GMP in κ-CN protruding hair is highly negatively charged, providing a strong repulsive steric stability that prevents casein aggregation and collapse of micelles. Chymosin action, during the production of natural cheese or RCN powder, the GMP is cut off [48], drastically diminishing the negative charge and changing the stability (steric) of casein micelle (κ-CN), leading to the collapse of the casein micelle’s structure and para-casein aggregation. This para-casein complex found in RCN powder and natural cheese is highly insoluble, whereas the casein obtained from the membrane process still has intact GMP and is soluble [9]. The RCN powder has higher pH and ash content, especially colloidal calcium phosphate, having Ca and P [49,50]. 

On the other hand, the intact casein obtained through a membrane system (UF or MF) contains casein micelles with GMP intact, making the casein micelle surface highly negatively charged, which helps it to maintain its integrity and soluble nature. Additionally, depending on the amount of diafiltration (DF) water used, the product from membrane systems may have reduced levels of soluble constituents such as SP, lactose calcium, and other minerals [8,9]. The addition of DF water during membrane processing helps in removing soluble constituents. When emulsifying salts are present, the highly negative casein micelle surface may inhibit or limit the native casein dispersion. This, along with the changed product matrix of UF and MF concentrates and powders, has a huge impact on the functionality. 

When insoluble rennet casein is used for IMC manufacturing, the small aggregates of insoluble para-casein remain even after addition and interaction with emulsifying salts. This helps to form a strong IMC structure after heating, mixing, and cooling during the manufacturing process, which provides a firmer texture during the melting of the long strands [8,9]. The action of TGase changes the surface properties of proteins (primarily caseins) present in MPC and MCC by crosslinking [8,9,18,19,38] and reduces negative charges on casein micelles [8,9]. The TGase action modifies the surface properties of the casein micelles [8,9,18,19,38]. The proteins from the membrane system change from soluble to insoluble [8,9,38], and the product can function similarly to RCN. The TGase treatment modified certain functional properties in MCC and MPC; however, it still could not provide the melt characteristics imparted by RCN. One of the reasons for the superior melt characteristics of RCN is better emulsification and protein texture formation. The TGase-treated samples had strong covalent bond formation, which may restrict the proper emulsion formation and cause meltability issues, as observed. Additional or changes in emulsifying ingredients may be necessary for TGase-treated powders.

Using a twin-screw cooker to manufacture IMC, similar hardness and measured meltability results in IMC compared with IMC made using RCN have been reported [8,9]. Increased RVA cooked viscosity, increased hardness, and restricted melt in PCP made with TGase-treated MCC (TGase used at 7 U/g of protein) [38]. By adding 0.04% TGase to milk, improvements in textural characteristics of mozzarella cheese have been reported [51]. Some have reported enhancement of mozzarella cheese protein structure using TGase [52]. A different texture in Kasher cheese was observed when TGase (0.75 U/g of protein) was used in the cheese curd with emulsifying salt [53]. In the model of PC samples made with RCN, as SP content increased, a decrease in meltability has been reported [54,55,56,57]. Hence, along with TGase action, serum proteins can cause melt restriction.

## 4. Conclusions

The IMC was manufactured using RVA and is useful in characterizing the ingredients on a small scale. When used in IMC formulation, the highest TGase concentration in MCC or MPC did not form an emulsion due to extensive crosslinking. The product formulation, processing conditions, and emulsifying salts could not overcome the strong covalent bonds formed by TGase at the highest concentration. The TGase action significantly affected the firmness and meltability of IMC samples. The serum proteins also affected the meltability measured using a modified Schreiber test. The IMC made from MCC had higher meltability measured using TT values and Schreiber melt test area compared with that made from MPC (with or without TGase). The primary IMC requirements for hardness and meltability changed in L-MCC and L-MPC after the TGase action. TGase-treated IMC samples (L-MCC and L-MPC) were similar to IMC samples produced using RCN in terms of hardness and viscosity during manufacture. However, the TT was significantly (*p* < 0.05) higher and meltability was restricted for TGase-treated samples compared to RCN samples. The TGase action in MPC and MCC restricts the meltability of the IMC. The TGase treatment could not match the meltability results provided by RCN, but it did modify certain functional properties in MCC and MPC. This study shows that TGase treatment alters the melt characteristics of MCC and MPC in IMC applications, and some properties can be matched with the IMC made using RCN.

## Figures and Tables

**Table 1:** Formulation and ingredient blend utilized to manufacture the 5 IMC ^1^ treatments in RVA ^2^.

	Imitation Mozzarella Cheese Treatment ^3^				
Ingredient	C-MCC	L-MCC	C-MPC	L-MPC	RCN
	% (*w*/*w*)				
Water	42.66	42.56	42.50	42.45	40.83
Lactic acid	0.80	0.80	0.80	0.80	0.80
Salt	1.00	1.00	1.00	1.00	1.00
Butter (unsalted)	24.71	24.70	24.92	24.90	25.89
Whey deproteinized	1.93	2.05	0.72	0.81	4.66
Citric Acid	0.50	0.50	0.50	0.50	0.50
Kasal	3.00	3.00	3.00	3.00	3.00
Treatment powder	25.39	25.39	26.55	26.54	23.33
					
Total	100.00	100.00	100.00	100.00	100.00

^1^ IMC = imitation mozzarella cheese treatment. ^2^ RVA = Rapid Visco Analyzer. ^3^ Imitation mozzarella cheese treatment: C-MCC = control—no enzyme, micellar casein concentrate powder; L-MCC = low enzyme, micellar casein concentrate powder; C-MPC = control—no enzyme, milk protein concentrate powder; L-MPC = low enzyme, milk protein concentrate powder; RCN = imitation mozzarella cheese treatment—rennet casein.

**Table 2:** Mean values (*n* = 3) of the proximate composition of the 5 IMC ^1^ treatments manufactured in RVA ^2^.

Parameters	Imitation Mozzarella Cheese Treatment ^3^				
	C-MCC	L-MCC	C-MPC	L-MPC	RCN
pH	5.65	5.65	5.65	5.63	5.64
Fat, %	20.99 ^a^	20.94 ^b^	20.98 ^ab^	20.96 ^ab^	21.01
Protein, %	19.98	19.95	19.97	19.95	20.06
Moisture, %	47.95	47.84	48.01	47.97	48.03

^a,b^ Means within the same row not sharing common superscript are significantly different (*p* < 0.05). ^1^ IMC = imitation mozzarella cheese treatment. ^2^ RVA = Rapid Visco Analyzer. ^3^ Imitation mozzarella cheese treatment: C-MCC = control—no enzyme, micellar casein concentrate powder; L-MCC = low enzyme, micellar casein concentrate powder; C-MPC = control—no enzyme, milk protein concentrate powder; L-MPC = low enzyme, milk protein concentrate powder; RCN = imitation mozzarella cheese treatment—rennet casein.

**Table 3:** Mean squares and *p*-values (in parentheses) of the 4 IMC ^1^ treatments manufactured in RVA ^2^.

Factors	df	RVA-Viscosity	TT ^3^	Change in Area	TPA-Hardness
Replication	2	58,606.64 (0.364)	5.94 (0.737)	1925.71 (0.818)	2,733,702.88 (0.037) *
Product Type	1	31,249.10 (0.454)	200.63 (0.017) *	158,224.26 (0.006) *	994,241.96 (0.190)
Enzyme Level	1	4,148,286.57 (<0.0001) *	207.22 (0.015) *	460,272.96 (0.0004) *	2,768,266.69 (0.049) *
Product Type × Enzyme Level	1	28,722.98 (0.472)	9.13 (0.508)	2319.63 (0.635)	3,658,420.01 (0.030) *
Error	6	48,708.38	18.46	9260.14	455,078.53

* Statistically significant at *p* < 0.05. ^1^ IMC= Imitation mozzarella cheese treatment. ^2^ RVA= Rapid Visco Analyzer. ^3^ TT—transition temperature.

**Table 4:** Mean values (*n* = 3) of the melted functional properties of the 4 IMC ^1^ treatments manufactured in RVA ^2^.

Parameters	Imitation Mozzarella Cheese Treatment ^3^			
	C-MCC	L-MCC	C-MPC	L-MPC
RVA-viscosity, cP	1650.0 ^c^ ± 267.2	2923.8 ^a^ ± 168.5	1849.9 ^b^ ± 272.8	2927.9 ^a^ ± 174.7
TT ^4^, °C	75.6 ^b^ ± 3.26	82.1 ^a^ ± 2.86	65.6 ^c^ ± 3.07	75.7 ^b^ ± 5.18
Change Area, mm	798.1 ^a^ ± 74.5	378.6 ^c^ ± 99.9	540.7 ^b^ ± 80.5	176.8 ^d^ ± 87.8

^a–d^ Means within the same row not sharing common superscript are significantly different (*p* < 0.05). ^1^ IMC = imitation mozzarella cheese treatment. ^2^ RVA = Rapid Visco Analyzer. ^3^ Imitation mozzarella cheese treatment: C-MCC = control—no enzyme, micellar casein concentrate powder; L-MCC = low enzyme, micellar casein concentrate powder; C-MPC = control—no enzyme, milk protein concentrate powder; L-MPC = low enzyme, milk protein concentrate powder. ^4^ TT—transition temperature.

**Table 5:** Mean values (*n* = 3) of the unmelted functional properties of the IMC ^1^ treatments manufactured in RVA ^2^.

Parameters	Imitation Mozzarella Cheese Treatment ^3^			
	C-MCC	L-MCC	C-MPC	L-MPC
Hardness, g	5852.4 ^a^ ± 1551.9	5708.7 ^a^ ± 870.0	4172.4 ^b^ ± 895.1	6237.3 ^a^ ± 804.1
Adhesiveness, g.s	−333.7 ^b^ ± 310.03	−168.4 ^a^ ± 186.11	−341.1 ^b^ ± 214.72	−201.1 ^ab^ ± 81.66
Springiness	0.23 ^b^ ± 0.05	0.43 ^a^ ± 0.05	0.12 ^c^ ± 0.02	0.21 ^b^ ± 0.06
Cohesiveness	0.17 ^b^ ± 0.02	0.19 ^a^ ± 0.03	0.13 ^c^ ± 0.01	0.15 ^b^ ± 0.02
Gumminess	972.6 ^a^ ± 346.9	1071.1 ^a^ ± 242.3	542.5 ^b^ ± 125.1	964.6 ^a^ ± 213.6
Chewiness	235.0 ^b^ ± 123.2	456.6 ^a^ ± 130.0	64.8 ^c^ ± 20.9	214.5 ^b^ ± 99.2
Resilience	0.031 ^b^ ± 0.00	0.041 ^a^ ± 0.01	0.025 ^c^ ± 0.01	0.031 ^b^ ± 0.00

^a–c^ Means within the same row not sharing common superscript are significantly different (*p* < 0.05). ^1^ IMC = imitation mozzarella cheese treatment. ^2^ RVA= Rapid Visco Analyzer. ^3^ Imitation mozzarella cheese treatment: C-MCC = control—no enzyme, micellar casein concentrate powder; L-MCC = low enzyme, micellar casein concentrate powder; C-MPC = control—no enzyme, milk protein concentrate powder; L-MPC = low enzyme, milk protein concentrate powder; RCN = imitation mozzarella cheese treatment—rennet casein.

**Table 6:** Mean values (*n* = 3) of the functional properties of the rennet casein IMC ^1^ and comparison with IMC treatments manufactured in RVA ^2^.

Parameters	Imitation Mozzarella Cheese Treatment ^3^				
	RCN	C-MCC	L-MCC	C-MPC	L-MPC
	Unmelted characteristics				
Hardness, g	6386.3 ± 728.87	NS	NS	NS	NS
Adhesiveness, g.s	−202.1 ± 86.20	NS	NS	NS	NS
Springiness	0.40 ± 0.09	*	NS	*	*
Cohesiveness	0.21 ± 0.02	NS	NS	*	*
Gumminess	1331.3 ± 270.49	NS	NS	*	NS
Chewiness	546.5 ± 208.07	*	NS	*	*
Resilience	0.04 ± 0.01	NS	NS	NS	NS
	Melted characteristics				
RVA-Viscosity, cP	3488.0 ± 498.53	*	NS	*	NS
TT ^4^, °C	57.5 ± 0.42	*	*	NS	*
Change Area, mm	1142.7 ± 25.69	*	*	*	*

* Statistically significant at *p* < 0.05; NS = non-significant. ^1^ IMC = imitation mozzarella cheese treatment; ^2^ RVA—Rapid Visco Analyzer. ^3^ Imitation mozzarella cheese treatment: C-MCC = control—no enzyme, micellar casein concentrate powder; L-MCC = low enzyme, micellar casein concentrate powder; C-MPC = control—no enzyme, milk protein concentrate powder; L-MPC = low enzyme, milk protein concentrate powder; RCN = imitation mozzarella cheese treatment—rennet casein. ^4^ TT—transition temperature.

## Data Availability

The original contributions presented in the study are included in the article, further inquiries can be directed to the corresponding author.
